# A global immune gene expression signature for human cancers

**DOI:** 10.18632/oncotarget.26773

**Published:** 2019-03-08

**Authors:** Yuexin Liu

**Affiliations:** ^1^ Department of Bioinformatics and Computational Biology, The University of Texas MD Anderson Cancer Center, Houston, Texas, USA

**Keywords:** immune response, immunogenicity, gene signature, human cancer, gene expression

## Abstract

**Background:**

Except for a few, the immune gene signatures for most cancer types are not available. We sought to identify a global immune gene signature that is applicable to all human cancers.

**Results:**

We identified an immune gene signature that was intimately correlated with tumor immune characteristics of human cancers and consisted of 382 genes indicative of different immune cell types. The T helper type 1 and 2 cell activation pathway was most significantly enriched in this global immune gene set, while transcription factors, such as SPI1 and STAT family members, were the top regulators of this gene signature. Skin cutaneous melanoma with higher expression of this immune gene signature had significantly longer survival than those with lower immune gene expression. Breast cancer patients with higher immune gene signature were significantly associated with advanced-stage.

**Methods:**

We analyzed the gene expression profiles of 10,062 tumor samples from 32 cancer types in The Cancer Genome Atlas Pan-Cancer data set. Hierarchical clustering analysis of previously-defined immune genes was performed to identify a pan-cancer immune gene signature. Pathway and upstream regulator analyses were used to identify significantly enriched signaling pathways and transcription factors. Kaplan-Meier analysis was used to evaluate the survival difference between dichotomic groups with different immune gene signatures. Correlation of immune gene signature with tumor stage was also examined.

**Conclusions:**

Our identified immune gene signature is applicable in human cancers and can be used to characterize tumor immunogenicity within and across cancer types. Clinical implication of this immune gene set warrants future investigation.

## INTRODUCTION

Immunotherapy is emerging as a promising cancer treatment and has revolutionized the mechanistic understanding of tumorigenesis. Inhibitors against CTLA-4, PD-1, and PD-L1 proteins are in clinical use and have been responsible for long-lasting responses in different types of cancer [1, 2]. However, the response rate varies in patients both within and across cancer types. Approaches to characterize immunotherapy responsivity are in dire need. Bioinformatics analysis of high-throughput gene expression profiling has enabled identification of clinically-relevant molecular subtypes and has enhanced our understanding of molecular abnormalities of cancer [3–6]. In particular, immune gene expression signatures have been identified to characterize tumor immunity and thus predict the response to immunotherapy [1, 7, 8]. In some instances, immune gene signatures have been used to infer the fraction of immune cells in tumor samples [9]. Recently, The Cancer Genome Atlas (TCGA) PanCanAtlas research group used immune gene sets to determine immune-related molecular subtypes [10].

Gene expression–based immune signatures or immune scores have been proposed to characterize the immune response in cancer cell lines or tumor samples [11]. However, many of these are derived from single-disease studies (referred to as local immune gene signatures), and their application is limited to one or a few cancer types [12]. Many other cancer types and especially rare tumors have no specifically-defined immune gene signatures, in some cases because of small sample sizes. Recently, TCGA, a collaborative project of the National Cancer Institute and the National Human Genome Research Institute, has carried out a large-scale multi-platform genomic and integrated PanCanAtlas study of more than 11,000 human tissue samples from 33 major cancer types [13]. In the context of pan-cancer tumors, how to identify a pan-cancer or, in other words, a global immune gene signature that is applicable to a wide array of human cancers remains a challenge. Identification of a universal (general) immune gene signature to comprehensively understand immune characteristics across human cancer may provide a better understanding of tumor immunogenicity in a broader sense and facilitate the development of novel immune targets for rare tumors.

In this study, we developed a new method that capitalizes on gene expression profiles of a large set of tumor samples, with mixed cancer types, to identify a pan-cancer immune gene expression signature that is applicable across a variety of human cancers, including rare tumors. By performing unsupervised clustering analysis, we identified a group of genes that were intimately correlated with tumor characteristics across 32 different cancer types and that was referred to as a global immune gene expression signature. We further showed that this gene signature consists of genes indicative of different immune cell types or cellular immune functions. We investigated signaling pathways and upstream regulators significantly enriched in this gene set, as well as the prognostic capability of this gene signature in patients with skin cutaneous melanoma (SKCM) and with breast cancer (BRCA). The enriched signaling pathway was also verified by cellular composition.

## RESULTS

### Identification of a global immune gene expression signature in human cancer

**Figure 1:**
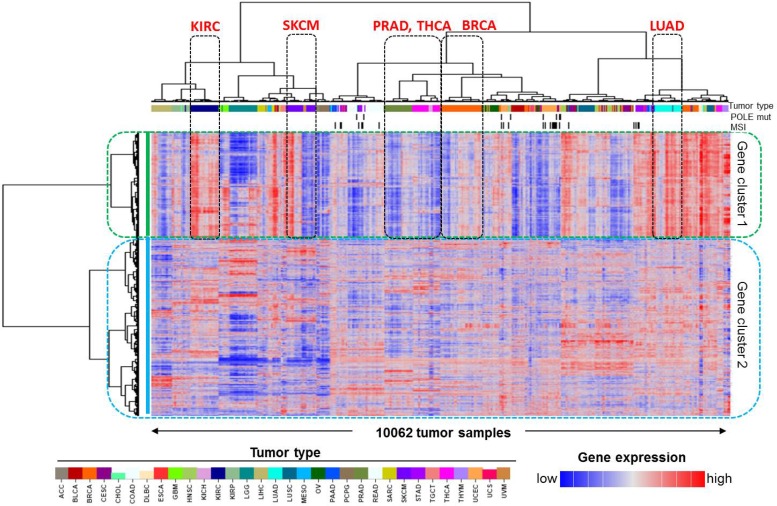
Identification of the pan-cancer immune gene expression signature in human cancer Unsupervised clustering analysis of 10,062 TCGA PanCanAtlas tumor samples and 1,031 previously reported immune-related genes revealed two groups of genes, namely, gene cluster 1 and gene cluster 2. The tumor types with apparently differential expression of cluster 1 genes, such as kidney renal clear cell carcinoma (KIRC), lung adenocarcinoma (LUAD), prostate adenocarcinoma (PRAD), and thyroid carcinoma (THCA), are indicated. SKCM and BRCA patients were split into two subgroups and are also indicated on the plot. The covariate bars on the top showed tumor types indicated by the color bar, MSI, and POLE mutation status, respectively. In the heatmap, red indicates high gene expression and blue indicates low expression.

To identify a global immune gene signature, we have employed an unbiased approach in this study and carried out an unsupervised hierarchical clustering analysis of 10,062 human cancer samples and 1,031 reliably measured immune genes (Figure 1). Similar to most of the other TCGA PanCanAtlas projects [13–15], the tissue-of-origin features provided the dominant signals for clustering of the tumor samples (columns in the heatmap). Slightly different from what we have previously done [3], in this study we were interested in the clustering of genes (rows in the heatmap), and selected the genes for clustering analysis by literature survey, rather than highly variable genes. Because genes with high expression variability were more likely confounded by tissue differentiation markers, we believe that cancer types did not have much impact on gene clustering (rows) as they did on clustering of tumor samples (columns). Moreover, because the tumor samples were grouped together largely by cancer types, we can therefore use the known tumor characteristics of those cancer types to determine biological functions of those identified gene clusters. It was apparent that the 1,031 genes were categorized into two major clusters, which are referred to as, gene cluster 1 (382 genes) and gene cluster 2 (649 genes). Compared to gene cluster 2, gene cluster 1 exhibited a clear and consistent expression pattern that was strongly correlated with tumor purity or leukocyte fraction. Kidney renal clear cell carcinoma (KIRC) had high expression of cluster 1 genes (Figure 1). Consistent with this result, KIRC was previously reported to have low tumor purity, with a median value of only 54% [16], and conversely, a relatively higher leukocyte fraction [10]. Similarly, lung adenocarcinoma (LUAD) also had high expression of cluster 1 genes, as indicated in the figure, as well as the second highest leukocyte fraction among the 33 PanCanAtlas cancer types [10]. Conversely, prostate adenocarcinoma (PRAD) and thyroid carcinoma (THCA) each had a relatively low expression of gene cluster 1. Consistent with this observation, these tumors also had a relatively low leukocyte fraction [10]. It has been previously reported that microsatellite instability (MSI) and POLE mutations were associated with immune activation [17, 18]. We next examined the relationship of MSI status and POLE mutation status with the identified immune gene signature. We obtained the POLE mutations from cBio portal [19] and MSI status data from the TCGA PanCanAtlas publications [14, 20]. It seemed that POLE mutated tumors were not grouped together, likely because POLE mutations have a low overall rate (~1.0%) in the entire PanCanAtlas cohort and, therefore, had a limited weight on clustering of tumor samples. MSI tumors were mainly from gastrointestinal/endometrial cancer, and the overall percentage was about 3.0%. Different from POLE mutations, MSI tumors appeared to group together, but exhibited opposite patterns with the reported immune gene signature. Some MSI tumors were associated with high immune signatures in contrast to other MSI tumors associated with a low immune signature. This observation is consistent with previous reports [21, 22], showing that MSI tumors were categorized into two groups and exhibited opposite immune profiles. On the other hand, gene cluster 2 exhibits much weaker correlation with tumor entities, suggesting that genes included in this cluster are likely disease-specific immune genes. Therefore, it is not trivial to apply these genes to characterize tumor immunity in different cancer types. As a result, we believe that gene cluster 1 is strongly associated with pan-cancer tumor microenvironment characteristics, representing a global immune gene expression signature for human cancer.

### Gene composition of the immune gene expression signature

Next, we sought to investigate in detail the biological functions of genes that compose the identified global immune gene signature (gene cluster 1). We first compared the new 382-gene signature with previously published immune gene sets indicative of specific immune cell populations [12]. We found that 79 (~20.7%) genes overlapped the previously reported T_Cell_cluster, 18 (~4.7%) genes overlapped the B_Cell_cluster, and 104 (~27.2%) genes overlapped the MacTh1_cluster, which consists of signatures indicating Th1 cells and macrophages. Among the remaining genes in the signature, 32 (~8.4%) genes were cytokines, chemokines, or their receptors [23], and 46 (~12.0%) additional genes were from the immune gene signature obtained from the literature [9]. The remaining 103 (30.0%) genes were reported to be generally involved in Gene Oncology (GO) biological processes related to immune functions.

### Pathway enrichment in the immune gene expression signature

**Figure 2:**
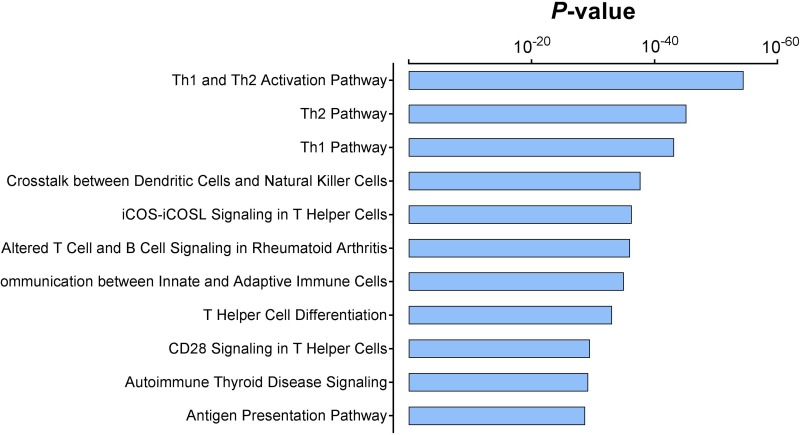
Significantly enriched signaling pathways in the immune gene expression signature The *P* values were assessed via Fisher's exact test and represented in logarithmic scale.

**Figure 3:**
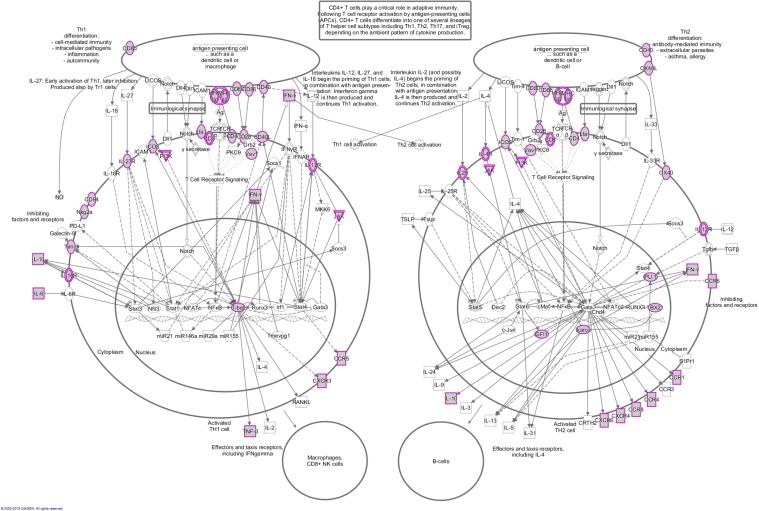
Schematic of the Th1 and Th2 activation pathway The gray nodes with purple borders represent genes that are included in the immune gene expression signature. The other genes shown are involved in this pathway, but not included in the signature.

To obtain a comprehensive overview of the identified immune gene expression signature, we used Ingenuity Pathway Analysis tools to associate this immune gene set with molecular pathways. The Ingenuity Knowledge Base, which includes all genes, was used as a reference set, and statistical significance was determined by Fisher's exact test. As anticipated, the most significantly enriched signaling pathways were all immune related, such as the Antigen Presentation Pathway, T Helper Cell Differentiation, and iCOS-iCOSL Signaling in T Helper Cells (Figure 2). Surprisingly, the first ranked pathway was the Th1 and Th2 Activation Pathway, with a statistical significance of less than 10^-55^, followed by the Th2 Pathway and the Th1 Pathway. The majority of the genes in the Th1 and Th2 Activation Pathway are located on the cellular membrane. In all, about 57 genes included in the immune gene expression signature are also involved in this signaling pathway (Figure 3).

### Upstream regulators of the immune gene expression signature

**Figure 4:**
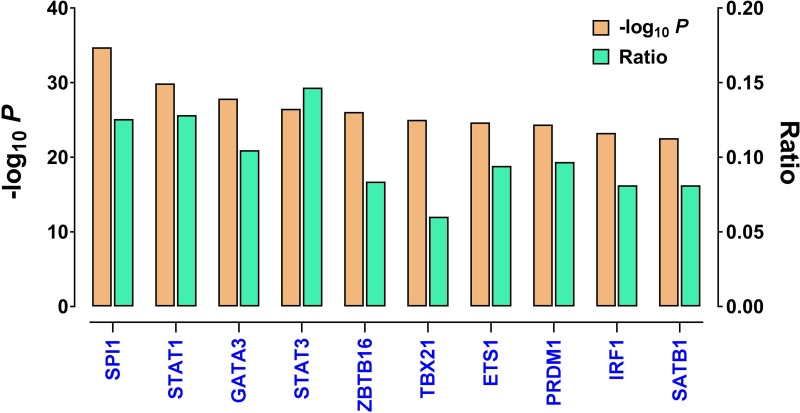
The top upstream transcription factors enriched in the immune gene expression signature, as identified by IPA Shown are *P* values (logarithmic scale) and the ratios of the number of predicted targets to the total number of genes included in the immune gene expression signature. The regulators are ranked in decreasing order of statistical significance.

Given that the immune gene expression signature is strongly associated with tumor immunogenicity, we next sought to investigate the underlying mechanism that leads to tumor immune activation. We first used the Ingenuity Pathway Analysis tool to identify potential nodes predicted to regulate the genes in this gene set, and statistical significance was assessed using Fisher's exact test. The results showed that *SPI1* was the most affected transcription factor in the upstream regulator rankings, based on its *P* value (*P* = 4.78 × 10^−35^). About 12.5% of the signature genes were predicted to be *SPI1*'s targets. In addition, STAT family members (such as *STAT1* and *STAT3*) were also in this significantly enriched transcription factor list (Figure 4). The other listed regulators included *GATA3, ZBTB16, TBX21, ETS1, PRDM1, IRF1,* and *SATB1*. Prominently, among these 10 most significantly enriched transcription factors, five (*SPI1, STAT1, STAT3, PRDM1,* and *IRF1*) have been recently reported to be master regulators of pan-immune networks [10].

### Survival analysis in SKCM patients with differentially expressed immune gene signatures

There are several reasons why we selected SKCM as an example in this study. Firstly, SKCM is one of a few cancer types that were previously reported to potentially respond to the immune checkpoint inhibitor therapy [1, 7]. Secondly, there was a known relationship between CTLA-4 expression and the overall survival of SKCM patients [8]. Thirdly, the SKCM cases happened to be split into two major clusters in the heatmap that had differentially expressed immune gene signature (Figure 1). Therefore we chose SKCM as a positive control for the reported immune gene expression signature.

**Figure 5:**
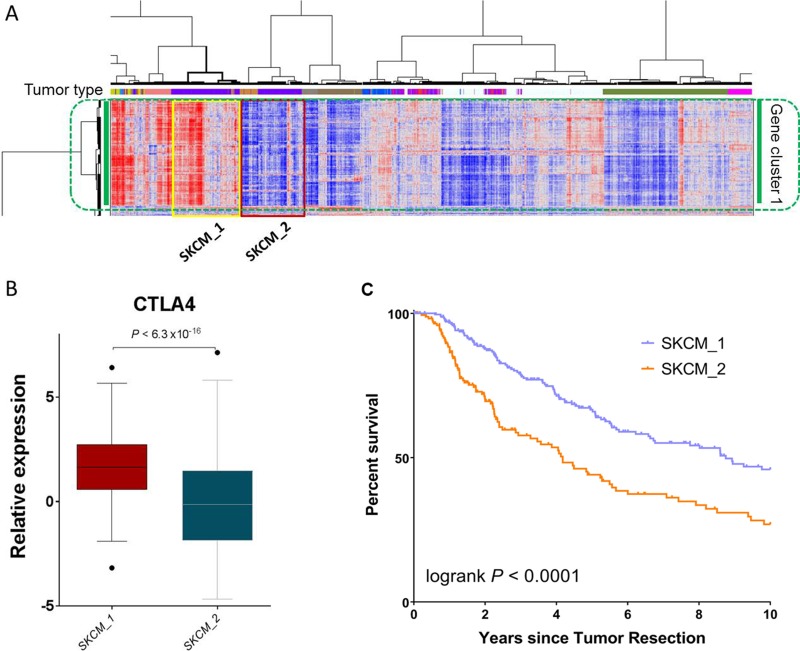
SKCM patients with differentially expressed immune gene signatures have significant survival differences (**A**) An enlarged portion of Figure 1, highlighting the two SKCM clusters, SKCM_1 and SKCM_2. Comparison of (**B**) *CTLA4* mRNA expression and **C**) patient overall survival between SKCM_1 and SKCM_2.

The two SKCM clusters were recapitulated in an enlarged version and determined on the basis of dendrogram branches (Figure 5A). Of note, the majority of uveal melanoma (UVM) patients were also clustered with SKCM cases, suggesting a similarity between these two cancer types. To alleviate the effect of UVM, we removed the UVM cases from the following analyses, naming the two SKCM-specific clusters SKCM_1 and SKCM_2. Specifically, SKCM_1 denotes the SKCM patients (*n* = 258) in the cluster with apparently higher immune gene expression signature, whereas SKCM_2 denotes the SKCM patients (*n* = 177) in the other cluster with an apparently lower immune gene expression signature. Consistent with this observation, *CTLA4* mRNA expression was significantly higher in the SKCM_1 patients than in the SKCM_2 patients (*P* < 6.3 × 10^-16^, Mann-Whitney test, Figure 5B). The Kaplan-Meier survival analysis showed that the SKCM_1 subgroup had significantly longer overall survival than the SKCM_2 subgroup (*P* < 0.0001, log-rank test, Figure 5C). These results are consistent with the anticipated clinical findings, suggesting the reliability of the immune gene signature identified in this study.

### Clinicopathological correlation of BRCA patients with differential immune gene signatures

**Figure 6:**
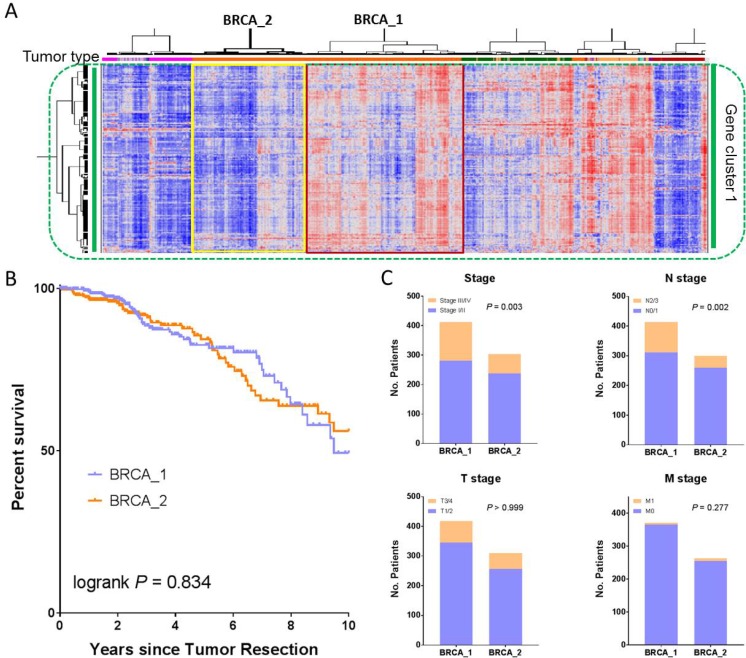
Clinicopathological correlation of BRCA patients with differentially expressed immune gene signatures (**A**) An enlarged portion of Figure 1, highlighting the two BRCA clusters, BRCA_1 and BRCA_2. Comparison of (**B**) patient overall survival and (**C**) both AJCC staging and TNM staging between BRCA_1 and BRCA_2.

Breast cancer (BRCA) was the most common cancer type in this study, representing 10.9% of the entire PanCanAtlas cohort (Table 1). Next we sought to examine the relationship between the reported immune gene signature and clinicopathological characteristics in breast cancer. As stated above, the BRCA cases were largely grouped together (Figure 1) and split into two major clusters with differential immune gene expression (Figure 6A). In accordance with the notation for SKCM, we named the cluster on the right with apparently high immune gene expression BRCA_1 (*n* = 418), and the cluster on the left with low immune expression BRCA_2 (*n* = 312). Different from SKCM, the two BRCA-specific clusters did not exhibit a significant difference in overall survival by Kaplan-Meier analysis (*P* = 0.834, log-rank test, Figure 6B). Although these two clusters were not significantly correlated with either T- (*P* > 0.999, Fisher's exact test) or M- (*P* = 0.277) stage, interestingly BRCA_1 had significantly more cases with Stage III or IV disease (*P* = 0.003) or cases with N2 or N3 disease (*P* = 0.002) (Figure 6C). This phenomenon is consistent with our recent report that immune activation in endometrial cancer was significantly enriched in the cases with a high-grade disease [21].

**Table 1:** Distribution of patient samples in the TCGA PanCanAtlas data set among the 32 cancer types that are sorted in descending order by their percentage in the cohort

Tumor type	Description	Patients	Percentage (%)
BRCA	Breast Invasive Carcinoma	1097	10.9
KIRC	Kidney renal clear cell carcinoma	533	5.3
UCEC	Uterine corpus endometrial carcinoma	532	5.3
HNSC	Head and neck squamous cell carcinoma	521	5.2
LUAD	Lung adenocarcinoma	516	5.1
LGG	Brain lower grade glioma	515	5.1
THCA	Thyroid carcinoma	505	5.0
LUSC	Lung squamous cell carcinoma	501	5.0
PRAD	Prostate adenocarcinoma	497	4.9
SKCM	Skin cutaneous melanoma	470	4.7
COAD	Colon adenocarcinoma	450	4.5
STAD	Stomach adenocarcinoma	418	4.2
BLCA	Bladder urothelial carcinoma	408	4.1
LIHC	Liver hepatocellular carcinoma	371	3.7
CESC	Cervical squamous cell carcinoma and endocervical adenocarcinoma	305	3.0
OV	Ovarian serous cystadenocarcinoma	305	3.0
KIRP	Kidney renal papillary cell carcinoma	290	2.9
SARC	Sarcoma	259	2.6
ESCA	Esophageal carcinoma	184	1.8
PCPG	Pheochromocytoma and paraganglioma	179	1.8
PAAD	Pancreatic adenocarcinoma	178	1.8
READ	Rectum adenocarcinoma	161	1.6
GBM	Glioblastoma multiforme	160	1.6
TGCT	Testicular germ cell tumors	134	1.3
THYM	Thymoma	120	1.2
MESO	Mesothelioma	87	0.9
UVM	Uveal melanoma	80	0.8
ACC	Adrenocortical carcinoma	79	0.8
KICH	Kidney chromophobe	66	0.7
UCS	Uterine carcinosarcoma	57	0.6
DLBC	Lymphoid neoplasm diffuse large B-cell lymphoma	48	0.5
CHOL	Cholangiocarcinoma	36	0.4

### Verification of Th cell pathway activation

**Figure 7:**
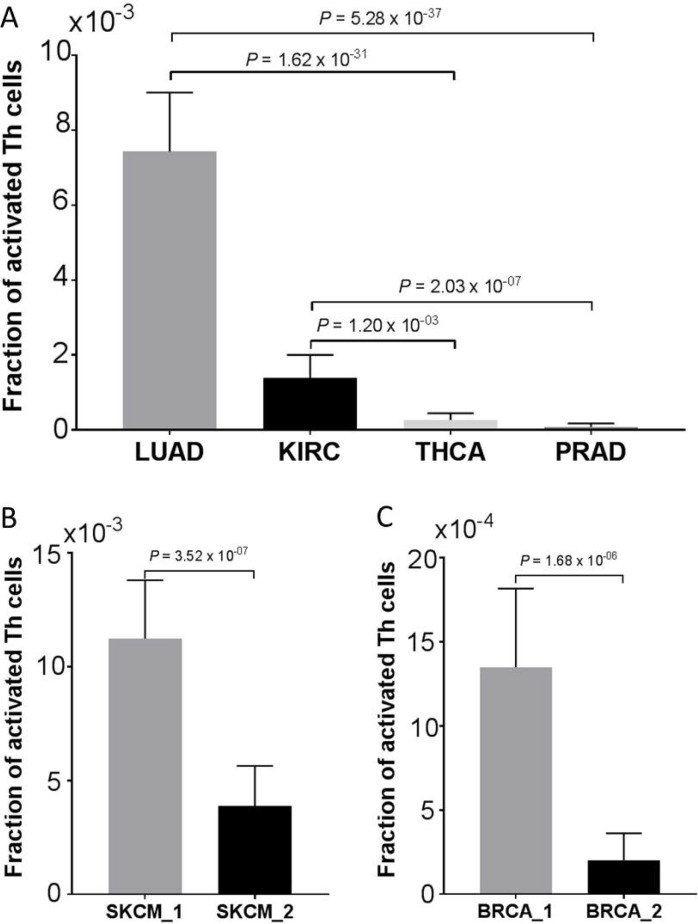
Verification of Th cells activation Correlation of estimated fractions of activated Th cells (**A**) among LUAD, KIRC, PRAD, and THCA, as discussed in Figure 1; (**B**) between the two SKCM clusters (SKCM_1 and SKCM_2), as depicted in Figure 5A; and (**C**) between the two BRCA clusters (BRCA_1 and BRCA_2), as depicted in Figure 6A. Data are presented as mean with 95% confidence intervals. The *P* values were calculated using a two-sided Man Whitney test.

We showed in the prior section that the Th1 and Th2 Activation Pathway was significantly enriched in the reported immune gene signature (Figures 3 and 4). To verify this result, we first obtained the Cell-type Identification By Estimating Relative Subsets Of RNA Transcripts (CIBERSORT) data from the TCGA Pan-Immune Response publication [10]. CIBERSORT is a cellular composition interference algorithm to determine the relative proportions of immune cells [24]. Among the CIBERSORT data, there was an immune cell type called “T.cells.CD4.memory.activated,” which represented activated T helper (Th) cells. We next compared the estimated fractions of this cell type (“T.cells.CD4.memory.activated”) among those cancer types, (i.e., LUAD, KIRC, PRAD, and THCA) as discussed earlier. LUAD and KIRC had a relatively high immune gene signature, as compared to PRAD and THCA (Figure 1). Consistent with this observation was that LUAD and KIRC also had a significantly higher fraction of activated T helper cells than PRAD and THCA (Figure 7A). In addition, we compared the estimated fractions of activated Th cells between the two SKCM-specific clusters (Figure 5A) and between the two BRCA-specific clusters (Figure 6A). Consistently, the clusters with apparently high immune gene signatures (i.e., SKCM_1 and BRCA_1) had a significantly greater fraction of activated Th cells than those clusters with low immune gene signatures (i.e., SKCM_2 and BRCA_2) (Figure 7B and 7C). Taken together, these data indicate that Th cell activation is strongly correlated with the reported immune gene signature, verifying the significant enrichment of the Th cell activation pathway in this immune gene set.

## DISCUSSION

Through hierarchical clustering analysis of previously defined immune genes and more than 10,000 TCGA tumor cases across 32 different cancer types, we identified a global immune gene expression signature, which we believe is broadly applicable across human cancers. In-depth statistical analyses show that this global immune gene signature is composed of genes indicative of different immune cell types and, as anticipated, significantly enriched in immune-related signaling pathways. We further show that *SPI1* and the STAT family are the top ranked modulators of this immune gene signature, representing key regulatory nodes of tumor immune response in human cancers. This identified immune gene signature can be used not only to characterize the immune response in cancer types with no available immune gene signature as of yet (most cancers), but to quantify the immune characteristics between different cancer types.

Compared to those proposed in the literature, the immune gene signature identified in this study has several advantages. First, it was generated through an unsupervised approach in a large patient population across a wide spectrum of cancer types; therefore our immune gene signature is unbiased and broadly applicable for human cancer. Most importantly, it fills the knowledge gap and can be used to characterize the immune response for the cancer types that lack available immune gene signatures, as of yet. Second, it is robust and scalable. The global nature of our immune gene signature enables us to evaluate or quantify the immune response for patients within a single cancer type or across different cancer types. For patients or cancer types with lower expression of immune gene signatures, an alternate therapy may be needed, although this hypothesis requires validation. Third, our immune gene signature helps understand immune response in a much broader sense and provides a generalized view of tumor immunogenicity that is common in all human cancers, although the clinical perspective of this gene signature deserves future investigation.

To our knowledge, we are reporting for the first time a global immune gene signature that can be used to characterize immunogenicity in a wide array of human cancers. The thus-identified gene signature is complex, in terms of gene content, containing biomarkers of cytotoxic T cells (*CD8A*), Treg cells (*FOXP3*), B cells (*CD79A*), macrophages (*CD68*), NK cells (*KLRC1*), dendritic cells (*LILRA4*), and neutrophils (*EVI2B*). Interestingly, PD-1 (gene symbol, *PDCD1*) and PD-L2 (gene symbol, *PDCD1LG2*), but not PD-L1 (gene symbol, *CD274*), are included in this global immune gene signature, likely because PD-L1 was expressed in intraepithelial immune cells, but not in tumor cells [17]. Another important immune checkpoint molecule, *CTLA-4*, is included in this signature, and overall survival was significantly longer in SKCM patients with higher *CTLA-4* expression than in those with lower expression. Anti-CTLA4 antibody has been used in clinical practice for treating metastatic melanoma.

This study provides new insights into tumor immune surveillance. It appears that the Th1 Pathway and Th2 Pathway are generalized signaling pathways to characterize the immune response across different cancer types. T helper type 1 (Th1) and T helper type 2 (Th2) cells are differentiated from naïve CD4+ T cells, following the activation of different cytokine signaling pathways. These two effector Th cells lineages play a significant role in orchestrating cell immunity [25]. Th1 cells activated by cytokines, such as IL-12 and IFNγ, provide immune response, particularly to intracellular pathogens. T-bet (gene symbol, *TBX21*) is the essential transcription factor for Th1 cell differentiation [26] and IFNγ production. Consistent with significant enrichment of the Th1 pathway, we found that *TBX21* was one of the top ranked transcriptional regulators of this immune gene expression signature. On the other hand, *GATA3*, also included among the top ranked transcription factors (Figure 6), drives the generation of Th2 cells [27], which are activated by IL-4, IL-5, and IL-13 and induce the humoral response against parasites. In addition to *TBX21* and *GATA3*, the STAT family members are among the top ranked transcription factors and also involved in the regulation of Th1 and Th2 cell differentiation.

SPI1 (transcription factor PU.1) was shown to be the most significantly enriched transcription factor that regulated the pan-cancer immune gene expression signature. Consistent with our findings, SPI1 was recently reported to be a key regulator of pan-immune networks through multiple different approaches [10]. Although it is beyond the scope of the current study, in the future we plan to perform a thorough interrogation on the relationship between this global immune gene signature and oncogenic pathways, copy number alterations, mutational load, and established clinicopathological characteristics, such as tumor metastasis, grade, and stage. Prognostic significance of the reported immune gene signature in all tumor types other than SKCM deserves systematic investigation to account for multi-hypothesis testing corrections.

In summary, our identified immune gene expression signature is applicable in a wide spectrum of human cancers and can quantify tumor immunity within or across cancer types. The clinical implication of this gene set and correlation with mutational load and oncogenic pathways warrants future investigation.

## MATERIALS AND METHODS

### Patient samples

The patient clinical annotation [28] and gene expression data [13] used in this study were obtained from the TCGA PanCanAtlas Research Network. This gene expression data set has merged gene expression data generated with different platforms and data generation centers. The clinical annotation contains patient information, such as tumor histology, tumor grade, disease stage, and overall survival duration. Overlapping of these two data types resulted in a total of 10,062 samples that had both gene expression and clinical data. The tumor tissues were obtained from 32 different cancer types (Table 1). The most common type was breast cancer, with more than 1,000 samples; the least common was uterine carcinosarcoma, with only 57 samples. Acute myeloid leukemia (the thirty-third PanCanAtlas cancer type) was not included in this analysis, because of missing clinical information.

### Immune-related gene sets

For first-order screening of informative genes used for clustering analysis, we started with a list of genes representative of biological characteristics that have been previously reported to be associated with immune response or immune suppression. We assembled this list by undertaking an extensive literature search and by using diverse resources, which were considered to be reliable and comprehensive [9, 12, 23, 29–31]. We first obtained the immune gene sets indicative of specific cellular immune populations from two different sources [12, 31]. We then obtained a list of genes associated with immune suppression, and a collection of genes for chemokines, cytokines, and their receptors [23]. We also included a 200-gene set that was highly specific to tumor T-cell infiltration [29] and an additional immune gene set [9]. To obtain an encompassing list of immune genes, we included the immune function genes identified from different treatment arms in clinical trials [30] and a list of immune metagene attractors derived from computational analysis [10, 32]. Of note, we did not include the Broad Institute molecular signatures data base (MSigDB), because the immune signatures in this database essentially cover the entire genome. In all, the combined gene list contained about 1,800 genes.

### Gene expression clustering analysis

Our clustering analysis was performed similar to what we did previously [14]. The immune gene expression profiling of the total 10,062 human cancer samples was further filtered to eliminate unreliably measured genes and to limit the clustering to relevant genes [33]. First, we filtered out genes that were duplicated in multiple sources or that were not present in the TCGA data set. We then removed genes that had missing expression values in any of the samples. Next, we filtered out genes that had small expression values (less than 0.5 reads per kilobase million (RPKM)) in at least one-third of the samples. Implementation of these filters resulted in 1,031 unique genes with reliably measured expression and more or less with immune relevance. The gene expression data were then median centered and log transformed. Next, we applied the hierarchical unsupervised clustering analysis with the preprocessed gene expression data. The distance metric was one minus the Pearson's correlation coefficient, and the Ward method was used as a linkage algorithm. This unsupervised approach clustered genes and identified two robust gene clusters. The two gene clusters and their gene expression patterns were viewed by using the next-generation clustered heat map (NG-CHM) tool developed at The University of Texas MD Anderson Cancer Center [15]. To further verify the identified immune gene signature, we also incorporated the MSI status and POLE mutations covariate bars in the heatmap. We obtained the POLE mutations from cBio portal [19] and MSI status data from the TCGA PanCanAtlas publications [14, 20]. POLE was commonly mutated in endometrial and colorectal cancer, while MSI tumors frequently occurred in endometrial, stomach, and colon cancer.

### Pathway analysis and upstream regulator analysis

Pathway analysis (Ingenuity Pathway Analysis (IPA)) was applied to identify the enrichment of signaling pathways in the immune gene expression signature. Upstream regulator analysis was used to identify transcription regulators predicted to regulate genes in this gene set.

### Analysis of patients with skin cutaneous melanoma

Unsupervised analysis classified the SKCM patients into two major subgroups. The first subgroup (referred to as SKCM_1), with apparent higher expression of immune gene signature, consisted of 258 SKCM patients; the second subgroup (referred to as SKCM_2), with apparent lower expression of immune gene signature, consisted of 177 SKCM patients. We then compared the gene expression difference in the *CTLA-4* gene between these two subgroups. After removing cases with missing survival data, we obtained 250 SKCM patients in the SKCM_1 group and 175 SKCM patients in the SKCM_2 group. The difference in overall survival between these two subgroups was then examined.

### Analysis of patients with breast cancer

The BRCA cases were categorized into major clusters. Consistent with the SKCM notation, BRCA_1 denotes the cluster with a high immune gene signature, and BRCA_2 denotes the cluster with low immune expression. After filtering out the cases from other entities, BRCA_1 was comprised of 418 BRCA patients and BRCA_2 consisted of 312 cases. In a similar manner, we performed a Kaplan-Meier survival analysis on these two BRCA-specific clusters. Correlation of these two clusters with tumor stage, as well as TNM staging, is also investigated.

### Statistical analysis

The statistical significance for both pathway analysis and upstream regulator analysis was assessed via Fisher's exact test. The correlation of BRCA-specific clusters with clinicopathological characteristics was examined via Fisher's exact test. We used the nonparametric Mann-Whitney test for comparison of CTLA-4 mRNA expression, as well as for comparison of estimated fractions of activated Th cells between the dichotomic groups. The Kaplan-Meier method was used to evaluate survival difference between these SKCM clusters, and statistical significance was assessed via log-rank test. All statistical tests were two-sided, and a *P* value of less than 0.05 was considered significant. The calculations and graphs were made with GraphPad Prism, version 7.03 (GraphPad Software, Inc., La Jolla, CA).

## Abbreviations

ACC: Adrenocortical carcinoma
BLCA: Bladder Urothelial Carcinoma
BRCA: Breast Invasive Carcinoma
CESC: Cervical Squamous Cell Carcinoma and Endocervical Adenocarcinoma
CHOL: Cholangiocarcinoma
COAD: Colon Adenocarcinoma
DLBC: Lymphoid Neoplasm Diffuse Large B-cell Lymphoma
ESCA: Esophageal Carcinoma
GBM: Glioblastoma Multiforme
HNSC: Head and Neck Squamous Cell Carcinoma
KICH: Kidney Chromophobe
KIRC: Kidney Renal Clear Cell Carcinoma
KIRP: Kidney Renal Papillary Cell Carcinoma
LGG: Brain Lower Grade Glioma
LIHC: Liver Hepatocellular Carcinoma
LUAD: Lung Adenocarcinoma
LUSC: Lung Squamous Cell Carcinoma
MESO: Mesothelioma
OV: Ovarian Serous Cystadenocarcinoma
PAAD: Pancreatic Adenomcarcinoma
PCPG: Pheochromocytoma and Paraganglioma
PRAD: Prostate Adenocarcinoma
READ: Rectum Adenocarcinoma
SARC: Sarcoma
SKCM: Skin Cutaneous Melanoma
STAD: Stomach Adenocarcinoma
TGCT: Testicular Germ Cell Tumors
THCA: Thyroid Carcinoma
THYM: Thymoma
UCEC: Uterine Corpus Endometrial Carcinoma
UCS: Uterine Carcinosarcoma
UVM: Uveal Melanoma
TCGA: The Cancer Genome Atlas
Th1 cell: T helper type 1 cell
Th2 cell: T helper type 2 cell
CTLA-4: cytotoxic T-lymphocyte associate protein 4.
